# Virulence and Resistance Mechanisms in Multidrug-Resistant *Acinetobacter baumannii*

**DOI:** 10.3390/pathogens15080798

**Published:** 2026-07-28

**Authors:** Priya Rajendran, Rameshkumar Marimuthu Ragavan, Renuka James, Bindu Dhanapal, Mullai Venkatachalam, Jeevarahini Reghupathy, Ramachandran Vignesh

**Affiliations:** 1 Department of Bacteriology, ICMR—National Institute for Research in Tuberculosis, Chennai 600031, India; rahini1@gmail.com; 2 Department of Microbiology, School of Life Sciences, Central University of Rajasthan, Ajmer 305817, India; mrrameshkumar@curaj.ac.in; 3 Department of Microbiology, The American College, Madurai 625002, India; renuka@americancollege.edu.in; 4 Department of Microbiology, Sree Balaji Medical College and Hospital, Chennai 600044, India; mail2bindhu@rediffmail.com; 5 Anderson Diagnostics & Labs, Chennai 600006, India; mullaisure@gmail.com; 6 Preclinical Department, Royal College of Medicine Perak, Universiti Kuala Lumpur, Ipoh 30450, Malaysia

**Keywords:** *Acinetobacter* spp., multidrug resistance, CRAB, virulence mechanism, treatment

## Abstract

*Acinetobacter baumannii*, a Gram-negative opportunistic bacterium in the ESKAPE group (*Enterococcus faecium*, *Staphylococcus aureus*, *Klebsiella pneumoniae*, *A. baumannii*, *Pseudomonas aeruginosa* and *Enterobacter* spp.), has emerged as a leading cause of nosocomial infections worldwide. It is known to possess diverse virulence traits and antimicrobial resistance, making it a critical priority pathogen on the World Health Organization’s 2024 Bacterial Priority Pathogens List. Carbapenem-resistant *A. baumannii* (CRAB) is currently endemic across several continents, with global carbapenem resistance exceeding 70% in healthcare settings and multidrug-resistant infections being associated with alarming mortality rates. This review comprehensively discusses the molecular underpinnings of *A. baumannii* pathogenesis and virulence, detailing the array of factors coordinated by complex regulatory networks. The convergence of this pathogen’s virulence and antimicrobial resistance traits, resulting in multidrug resistance, leaves clinicians with only a handful of therapeutic options. The review also discusses upcoming therapeutic strategies, including phage therapy, antimicrobial peptides, monoclonal antibodies, photodynamic therapy, and vaccine candidates in the pipeline. While emerging therapeutics show promise, several challenges remain, and integrated approaches are warranted to efficiently combat *A. baumannii*’s virulence and resistance armamentarium.

## 1. Introduction

The emergence and spread of antimicrobial resistance (AMR) is undoubtedly a global public health hazard with grave consequences for morbidity, mortality, and the economy [1]. Antibiotic-resistant infections have been estimated to cause over 1.2 million deaths globally in 2019 [2]. *A. baumannii* is one of the most clinically relevant species, and is associated with increased virulence and multidrug resistance [3]. It is commonly associated with hospital-acquired infections, particularly among critically ill patients, those admitted to intensive care units (ICUs), and individuals receiving invasive procedures such as mechanical ventilation or catheterization. Its remarkable ability to survive under harsh environmental conditions and acquire resistance determinants has contributed to its persistence in hospitals and rapid global dissemination. The increasing prevalence of multidrug-resistant (MDR), extensively drug-resistant (XDR), and carbapenem-resistant strains has become a major public health concern. *A. baumannii* is classified among the ESKAPE organisms (*Enterococcus faecium*, *Staphylococcus aureus*, *Klebsiella pneumoniae*, *A. baumannii*, *Pseudomonas aeruginosa*, and *Enterobacter* spp.). This group of organisms represents a major global threat to human health and poses significant therapeutic challenges due to the rapid emergence of AMR [1]. In response to the AMR threat, WHO, as part of its global action plan, developed an updated Bacterial Priority Pathogens List (BPPL) in 2024. Among the three priority tiers of BPPL (critical, high, and medium), carbapenem-resistant *A. baumannii* is listed under the critical tier [4]. *A. baumannii* is a particularly notable pathogen in this group, owing to its survival strategies in challenging environments like those of hospitals and its remarkable ability to accumulate resistant markers. These features enable the pathogen to cause life-threatening and difficult-to-treat infections, including hospital-acquired and ventilator-associated pneumonia (HAP, VAP), urinary tract infections, meningitis, bacteremia, gastrointestinal infections and wound infections [1,5,6]. The prevalence of multidrug-resistant (MDR) *Acinetobacter* strains in patients with HAP and VAP is about 80%, with an alarming overall mortality rate of about 56% [7]. Earlier, carbapenems were considered the first-line treatment for MDR *A. baumannii* strains. However, with the increasing incidence of carbapenem resistance, polymyxins are widely used despite their systemic toxicities [8,9]. To make matters worse, *A. baumannii* strains have been reported to be extensively drug-resistant (XDR), resistant to three or more classes of antimicrobials, and pan-drug-resistant (PDR), resistant to polymyxins and tigecycline [1,10,11]. This review aims to comprehensively examine the multifaceted aspects of *A. baumannii*, including its epidemiology, virulence mechanisms, antimicrobial drug resistance mechanisms, pathogenesis, and emerging therapeutic options in the pipeline.

## 2. Brief Epidemiology and Global Clinical Impact of *Acinetobacter baumannii*

The global epidemiology of *A. baumannii* reveals it as a highly adaptable pathogen that has successfully established itself across diverse healthcare systems worldwide, yet with notable geographic variations posing both universal and region-specific challenges. Globally, *A. baumannii* is estimated to account for over 300,000 deaths annually [12] and approximately 1 million cases per year [13], imposing significant strain on healthcare systems through prolonged hospitalizations, increased readmission rates (17.8% for resistant strains), and escalating costs [14]. According to comprehensive datasets from the WHO Global Antimicrobial Resistance and Use Surveillance System (GLASS), carbapenem-resistant *A. baumannii* (CRAB) has become endemic in healthcare settings across much of Latin America, the Mediterranean region, Asia and Africa [15]. Complementing these surveillance data, the Global Research on Antimicrobial Resistance (GRAM) Study identified CRAB as one of the leading contributors to AMR-associated mortality worldwide, with crude mortality rates for invasive infections commonly ranging from 30% to 75%, depending on the infection type and patient population [2]. The substantial global burden of CRAB underscores its recognition as a critical priority pathogen for antimicrobial development and infection control. Global surveillance conducted between 2016 and 2018 examined 4320 clinical *A. baumannii* isolates and found particularly high levels of carbapenem resistance. Meropenem susceptibility was lowest in Africa and the Middle East (17.2%), indicating that nearly 83% of isolates were resistant. Low susceptibility was also reported in Latin America (19.6%), Asia-Pacific (31.4%), and Europe (33.8%), while North America showed the highest susceptibility rate (63.6%). Resistance to other commonly used antibiotics, including amikacin and levofloxacin, followed a similar trend, with the highest resistance rates being observed in Africa/Middle East and Latin America [16]. According to reports from the WHO and the European Centre for Disease Prevention and Control (ECDC) in 2020, 35 of 38 surveyed countries reported carbapenem resistance rates exceeding 50% in Acinetobacter species, particularly in Southern and Eastern Europe. CRAB prevalence rates were reported to be above 70–90% in Greece and Italy, 40–65% in Spain [17]. During the COVID-19 pandemic, a multicenter study from intensive care units in Italy found that 19% of critically ill COVID-19 patients became colonized or infected with CRAB within an average of 10 days of ICU admission, and the mortality rate among these patients reached 67% [18].

In high-prevalence regions, such as India, *A. baumannii* causes 22.3% of hospital-acquired pneumonia cases, with carbapenem resistance at >70% and multidrug-resistant strains at 22.3%. A similar resistance pattern is also observed in China, with a higher prevalence range of 35.7–52.7% among hospital-acquired pneumonia cases [19]. Sharma et al. (2025) reported overall mortality rates of 30–75% for multidrug-resistant strains, with infections primarily affecting critically ill, ventilated patients in intensive care settings [20].

Molecular epidemiology has revealed the dominance of international clonal lineages (ICs), identified through multilocus sequence typing (MLST). Initially limited to three major clones (IC1–IC3), the classification has recently expanded to at least nine established clones (IC1–IC9), with emerging variants such as IC10. Among these, IC2 remains the most globally disseminated and clinically significant lineage, accounting for approximately 63% of CRAB isolates worldwide [21]. Regional variation exists, with IC5 predominating in parts of Latin America and newer clones emerging in the Middle East.

Collectively, these findings underscore that *A. baumannii* represents a critical global health threat, characterized by high mortality and an escalating burden of antimicrobial resistance.

## 3. Pathogenesis of *A. baumannii*

### 3.1. Primary Stages of Host Surface Colonization

The pathogenesis of *A. baumannii* is initiated by a highly coordinated, multi-step process through which the pathogen establishes itself on host tissues and indwelling medical devices. This pathogenic phase relies on an intricate interplay between initial sensing, electrostatic interactions and early-stage molecular adherence factors:

Sensing and initial attachment: Upon encountering a surface, *A. baumannii* utilizes mechanosensation to detect physical contact, triggering immediate downstream transcriptional changes. Initial reversible attachment is mainly brought about by non-specific physicochemical forces that overcome the electrostatic repulsion between the bacterial outer membrane and host cell surfaces [22].

Role of outer membrane components: The transition to irreversible attachment is mediated by specific cell-surface components, primarily by Outer Membrane Protein A (OmpA), a highly conserved structural porin that interacts with host epithelial cells, fibronectin and mucosal layers. In addition, OmpA plays a role by actively inducing host cell cytotoxicity and mitochondrial dysfunction, establishing a clear link between early colonization and tissue damage [5].

Surface hydrophobicity and capsular modulation: The success of early colonization greatly relies on the composition of the pathogen’s capsular polysaccharide. While a thick capsule can protect the bacterium from acute host immune clearance, dynamic modulation of capsule thickness allows the exposure of underlying surface adhesins, augmenting surface hydrophobicity and attachment efficiency on a wide range of anatomical locations.

### 3.2. Biofilm Architecture and Surface Dynamics

The transition from a motile state to a sessile lifestyle represents a primary defensive adaptation of *A. baumannii*, driving its long-term persistence on abiotic surfaces and host tissues. This architecture is governed by a well-established structural hierarchy:

Surface mobility and microcolony priming: Before the formation of a mature biofilm matrix, *A. baumannii* coordinates surface-associated and twitching motility via Type IV pili. This process is regulated by complex environmental systems. The structural variations in minor pseudo pilins such as *pilA* dictate functional specialization, transitioning the cell from an active state to a surface-anchored adherent stage [23,24].

The chaperone-usher System (Csu), comprising the csuA/BABCDE gene cluster, coordinates the assembly of pili that are essential for initial microcolony formation on abiotic medical surfaces [22]. The functional importance of the Csu system is strongly supported by evidence showing that its disruption consistently renders the strains biofilm-deficient, confirming it as a highly conserved mechanism [5,22].

Biofilm-associated proteins (BAP and BLP): Once initial attachment occurs, high-molecular-weight surface proteins like BAP stabilize the mature biofilm structure, facilitating the formation of intricate three-dimensional turret structures and biovolume. Unlike the highly conserved Csu system, BAP expression exhibits significant strain-dependent variation, with higher expression levels strongly correlating with hypervirulent clinical lineages [22].

Surface modulators and transcriptional regulation: The coordination of these structural elements with emerging surface modulators is fine-tuned by global regulators and catabolic pathways that modulate biofilm density in response to local nutritional and metabolic stress [22,24].

### 3.3. Cellular Adaptation and Intracellular Persistence

Beyond physical surface biofilms, *A. baumannii* employs several unique physiological strategies to survive within the hostile host environment, transitioning from an extracellular pathogen to a facultative intracellular threat.

#### 3.3.1. Intracellular Survival and Phagosomal Evasion

Although historically regarded as an extracellular pathogen, accumulating evidence indicates that many clinically relevant strains of *A. baumannii* can invade, survive and persist within host macrophages and epithelial cells [25,26]. Following internalization, *A. baumannii* can evade host-mediated degradation by interfering with phagosome−lysosome fusion and delaying phagosomal maturation, thereby reducing exposure to lysosomal enzymes and acidic pH [25]. Furthermore, the bacterium modulates multiple host signalling pathways, including those regulating autophagy, inflammation and cell death, thereby promoting intracellular persistence. Subsequently, the bacterium neutralizes host-derived reactive oxygen species (ROS) via the coordinated production of catalase and superoxide dismutase enzymes [25,26].

#### 3.3.2. Metabolic Adaptation

*A. baumannii* exhibits remarkable metabolic flexibility that enables adaptation to diverse host microenvironments. Depending on oxygen availability, the bacterium can switch from aerobic respiration to anaerobic metabolic pathways, facilitating survival under oxygen-limited conditions during infection. Under nutrient-limited conditions, it can utilize alternative carbon and energy sources, including host-derived fatty acids and amino acids, to sustain its metabolism. This metabolic adaptability promotes survival in nutrient-deficient environments, including host cells and on medical devices, thereby contributing to persistent infections and reduced susceptibility to antimicrobial therapy [25].

### 3.4. Genetic Plasticity and Phenotypic Heterogeneity

#### 3.4.1. MGE-Driven Genetic Plasticity

*A. baumannii* possesses a highly plastic genome shaped by horizontal gene transfer (HGT), genomic rearrangements and a diverse repertoire of mobile genetic elements (MGEs), including plasmids, transposons, and integrons [27,28,29]. These MGEs mediate the acquisition and dissemination of antimicrobial resistance genes and facilitate adaptation to changing environmental conditions and antibiotic selection pressures [28,29]. Together, these mechanisms enable rapid genetic diversification, contributing to the persistence and global emergence of multidrug-resistant *A. baumannii* lineages. The bacterium has the potential to undergo genomic rearrangements and HGT, which confer genetic plasticity alongside mobile elements, enabling it to respond quickly to new antibiotics and other challenging conditions [27,28,29].

#### 3.4.2. Persister Cell Dynamics

In contrast to genetically acquired resistance, A. baumannii forms transient persister cells that exhibit phenotypic tolerance to lethal antibiotic concentrations without acquiring resistance-conferring mutations. These cells can arise through metabolic downregulation and stress-response pathways, although the molecular mechanisms governing their formation remain incompletely understood [30,31]. Persisters survive antimicrobial treatment by entering a dormant state within biofilms and other protected host niches, then resuming active growth upon the subsidence of antibiotic pressure [30]. Consequently, they contribute to chronic and recurrent infections and may increase the likelihood of emergence of resistance during subsequent bacterial replication [30,31].

## 4. Virulence Mechanisms of *A. baumannii*

**Figure 1:**
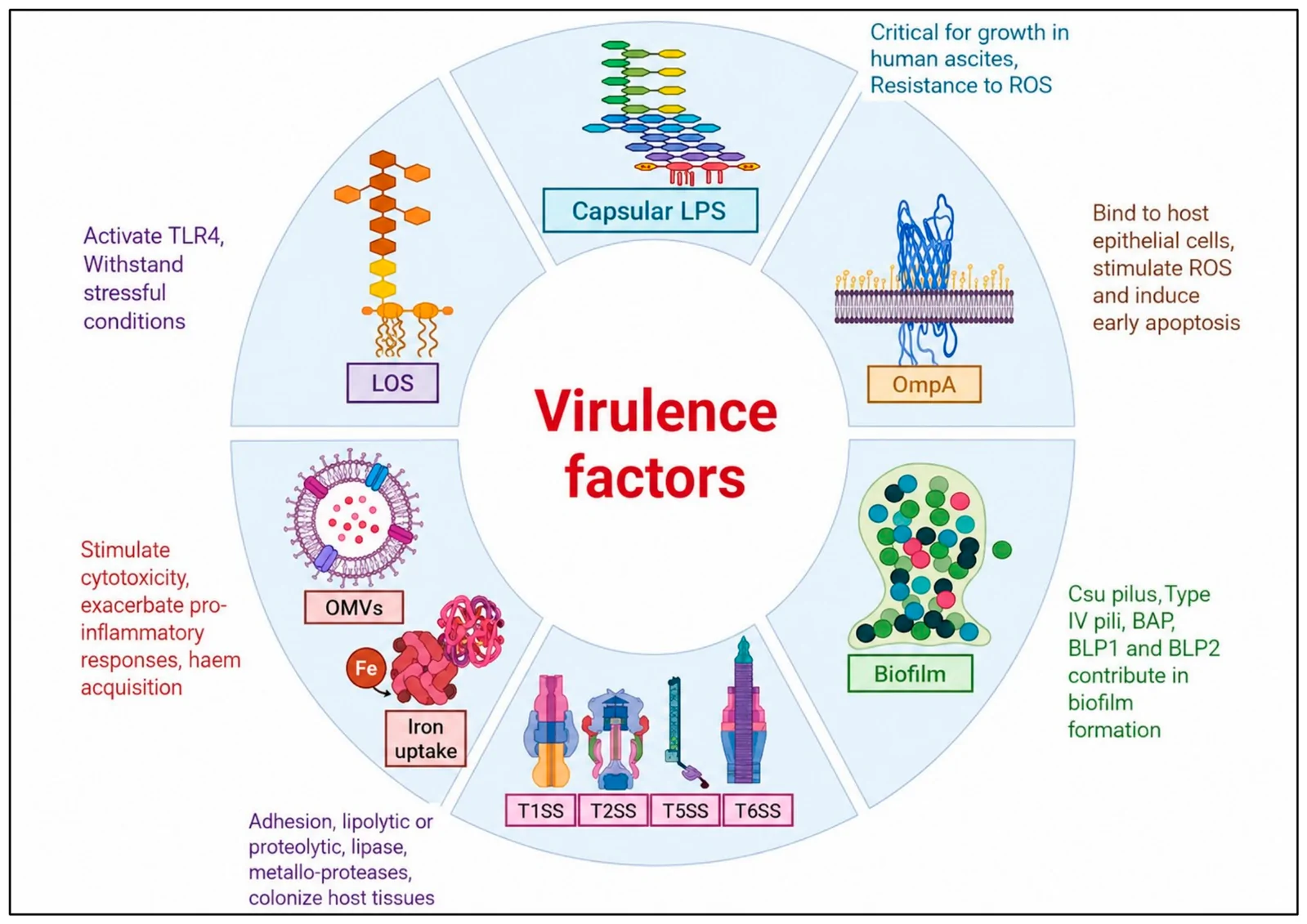
Virulence factors associated with *A. baumannii*. LOS: Lipooligosaccharide; TLR4: Toll-Like Receptor Type IV; LPS: Lipopolysaccharide; ROS: Reactive Oxygen Species; OmpA: Outer Membrane Protein A; BAPs: Biofilm-Associated Proteins; BLPs: Bap-Like Proteins; T1SS: Type 1 Secretion System; T2SS: Type 2 Secretion System; T5SS: Type 5 Secretion System; T6SS: Type 6 Secretion System; Fe: Iron; OMVs: Outer Membrane Vesicles. (Source: Image created using references [30,32].).

Several important virulence factors help *A. baumannii* to survive, spread, and resist host defences. Its outer membrane protein A (OmpA) promotes adherence to epithelial cells and induces host cell death, while biofilm formation on medical devices and hospital surfaces enhances persistence and antibiotic tolerance. The bacterium’s production of capsular polysaccharides protects against phagocytosis and desiccation. Iron acquisition systems, such as siderophores, help the organism obtain essential nutrients within the host, and lipopolysaccharides contribute to inflammation and immune evasion. In addition, secretion systems, phospholipases, and pili aid in colonization, tissue damage, motility, and establishment of infection, thereby contributing to the success of *A. baumannii* as a multidrug-resistant healthcare-associated pathogen (Figure 1).

### 4.1. Core Surface Components and Outer Membrane Architecture

#### 4.1.1. Outer Membrane Proteins (OmpA)

OmpA remains a well-established virulence determinant of *A. baumannii* pathogenicity, with its critical role being demonstrated in mammalian infection models [33]. In addition to functioning as a structural porin, it serves as a key driver of host cell attachment, microvascular endothelial cell damage and mitochondrial dysfunction, ultimately leading to apoptosis [33,34]. As a virulence factor, it is a promising therapeutic target since it is highly conserved across both virulent clinical isolates and laboratory strains. Monoclonal antibodies and small molecule inhibitors that target OmpA are being extensively investigated as a way to inhibit host–cell adherence [34].

#### 4.1.2. Capsular Polysaccharides

The capsule of *A. baumannii* serves as an important protective barrier against host nutritional and humoral immunity. Evidence from several animal models indicates the pivotal role of capsule expression levels in dictating serum resistance and systemic survival by preventing complement-mediated opsonization [27,35]. However, the capsule demonstrates strain-specific heterogeneity driven by a highly variable chromosomal K-locus. Hypervirulent lineages such as global clone 2 strains possess distinct capsular structures that yield hyper-mucoid phenotypes [35]. While capsule is a well-proven virulence factor, its structural variability lowers its utility as a universal vaccine target.

#### 4.1.3. Lipopolysaccharides (LPS) and Lipooligosaccharide (LOS)

The Lipid A moiety of *A. baumannii* LPS/LOS is highly conserved and serves as a powerful trigger for systemic inflammatory response syndrome (SIRS) via TLR4 activation [36]. *A. baumannii* is unusual among Gram-negative pathogens in its ability to survive the complete loss of LPS under colistin selective pressure [34,36]. This illustrates a physiological trade-off where colistin-resistant, LPS-deficient mutants display a marked reduction in fitness and in vivo virulence [36]. Hence, inhibition of LPS biosynthesis through LpxC represents a potential therapeutic strategy because disruption of lipid A synthesis compromises outer membrane integrity and may enhance bacterial susceptibility to host immune clearance [36,37].

#### 4.1.4. Pili and Fimbriae

Unlike OmpA or capsule, individual pili systems demonstrate significant functional redundancy [34]. The chaperone-usher (Csu) pili system is essential for abiotic glass and plastic colonization, while Type IV pili mediate biotic mucosal tissue attachment and twitching motility [33,34]. While in vitro genetic deletion studies clearly exhibit these phenotypes, experimental studies indicate that disruption of a single pili system does not completely abolish virulence, suggesting functional overlap among multiple adhesins and colonization factors [33]. Consequently, the therapeutic utility of targeting individual pili remains uncertain because of this functional redundancy.

### 4.2. Secreted Factors, Vesicles and Intracellular Communication

#### 4.2.1. Outer Membrane Vesicles (OMVs)

OMVs represent a conserved, highly active secretory mechanism utilized by A. baumannii to deliver a concentrated array of virulence factors (including OmpA, proteases and carbapenemases) directly into host tissues and competing bacteria [38,39]. Experimental studies have shown that OMVs contribute to tissue injury and proinflammatory responses in vitro and in animal models. OMVs are being explored as an emerging area of interest for outer-membrane targeted therapeutics because their production can be upregulated under antibiotic or environmental stress [38,39].

#### 4.2.2. Secretion Systems (T6SS and T4SS)

The Type VI Secretion System (T6SS) is supported by experimental studies as a key tool for inter-bacterial competition. However, it is of interest that expression of T6SS is highly strain-dependent, with many clinical isolates exhibiting transcriptional repression mediated by plasmid-associated regulatory mechanisms [40]. In contrast, the Type IV Secretion System (T4SS) primarily facilitates horizontal gene transfer through conjugative plasmid transfer, contributing to the dissemination of antimicrobial resistance rather than directly mediating host cell cytotoxicity [40,41].

#### 4.2.3. Phospholipases (PLC and PLD)

Phospholipases C and D contribute to host cell membrane disruption and cell lysis in vitro [42,43]. However, their contribution to human disease severity remains incompletely defined, as current evidence is derived primarily from in vivo studies and experimental animal models [43]. Compared with major virulence determinants such as OmpA, capsule polysaccharides and biofilm-associated factors, phospholipases appear to play a more supportive role in pathogenicity, although their precise contribution likely varies among strains and infection models [42,43].

#### 4.2.4. Quorum Sensing

**Figure 2:**
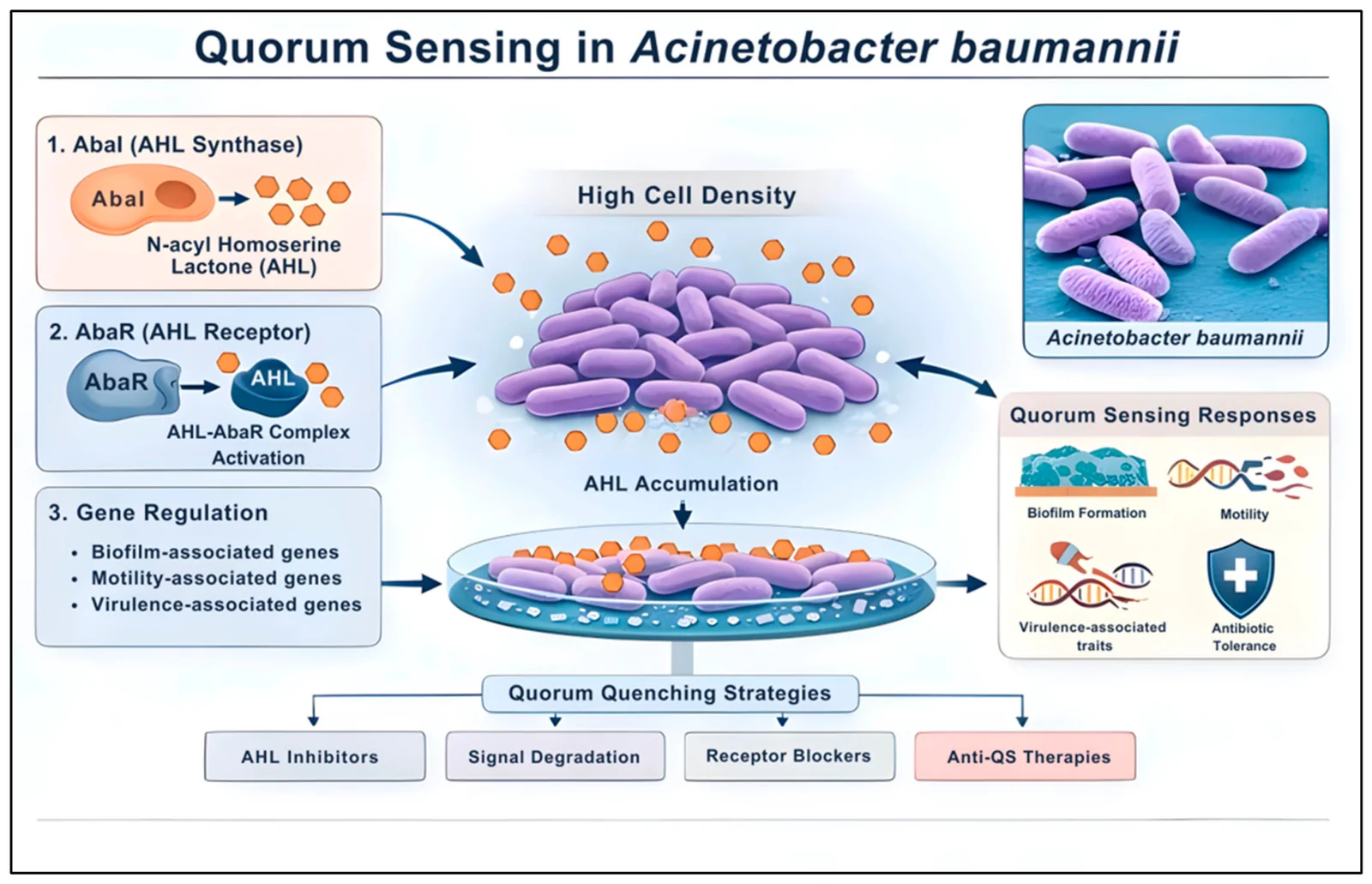
Schema of the QS System of AbaI and AbaR. As bacterial density increases, synthesised AHL accumulates; AHL binds to the receptor protein AbaR, forming a complex that activates the expression of genes involved in biofilm production, virulence factors, motility, and antibiotic resistance/tolerance. Quorum quenching involves strategies that inhibit AHL from promoting bacterial communication and ultimately pathogenicity. Examples include AHL inhibitors, AHL-degrading enzymes, receptor blockers, and anti-QS therapies. (Source: Image created using references [45,47].).

Quorum sensing (QS) and nucleotide second messengers (NSMs) represent interconnected regulatory networks that govern physiological adaptation and virulence expression in *A. baumannii* [44,45]. The primary quorum-sensing system relies on the LuxI/LuxR-type AbaI/AbaR system. The autoinducer synthase AbaI synthesizes long-chain N-acyl homoserine lactones (AHLs) which, upon reaching a critical density-dependent threshold, bind the cognate receptor AbaR to coordinate the expression of genes involved in surface attachment, motility and biofilm maturation [46] (Table 1, Figure 2).

While these signaling cascades are extensively documented in vitro, their overall contribution to in vivo virulence remains an area of ongoing critical evaluation, as clinical isolates possess multiple overlapping regulatory pathways that may partially compensate for disruption of quorum sensing under host conditions. Consequently, interfering with these pathways through ‘quorum quenching’—using AHL synthesis inhibitors, receptor antagonists, or enzyme-mediated degradation—presents an attractive anti-virulence strategy. By attenuating pathogenicity without directly arresting bacterial viability, these emerging therapies minimize the selective pressure for traditional resistance mutations, although their clinical efficacy as standalone treatments requires further in vivo validation (Figure 2).

## 5. Regulation of Virulence Mechanisms of *A. baumannii*

The virulence mechanisms of *A. baumannii* are intricately regulated by a sophisticated, multi-layered system that includes two-component systems (TCSs), quorum sensing (QS) networks, RNA-binding proteins, and small non-coding RNAs (sRNAs). These elements of the regulatory architecture respond to environmental signals, thereby coordinating the expression of secretion systems, adhesins, and biofilm determinants.

### 5.1. BfmRS Two-Component System

The BfmRS TCS serves as the key hub that controls both the resistance and virulence mechanisms of *A. baumannii*. Phosphorylation of BfmR is the key activating step leading to the response regulator directly influencing gene expression. BfmR has been shown to induce tolerance in the bacterium to oxidative stress and desiccation, and is also implicated in modulating resistance to β-lactams [50,51]. BfmR has also been shown to help the bacterium form a pellicle—a tightly packed biomass in the culture media, thereby modulating cooperative behaviour against closely related strains. This pellicle formation enables the bacterium to acquire a favourable ecological niche and access high concentrations of oxygen and nutrients, thereby making it an efficient opportunistic pathogen. BfmR is also involved in the upregulation of the CsuA/BABCDE operon, which is responsible for biofilm formation [52]. BfmR can also negatively regulate the AbalR quorum-sensing system, thereby revealing a coordinated, multilayered regulatory approach. BfmRS has been shown to support augmented tolerance to antimicrobials such as carbenicillin and fluoroquinolones. It is also known to modulate the production of outer membrane vesicles and are implicated in enabling motility in the bacterium [53].

### 5.2. Quorum-Sensing (QS) System

A third gene, *abaM,* located in the QS locus of *A. baumannii* between *abaR* and *abaI*, encodes an RsaM-type regulator that modulates quorum sensing. [54]. Its disruption has been shown to enhance the production of N-acyl homoserine lactone (AHL). Studies have shown that mutation of *abaM* increased surface motility and biofilm development, but attenuated *A. baumannii’s* virulence [48].

### 5.3. RNA-Binding Proteins and sRNAs

CsrA is an RNA-binding protein with regulatory functions and recent research indicates that CsrA acts as a key positive regulator of the switch from virulent to avirulent (AV-T) sub-populations of *A. baumannii* [55]. The activity of CsrA is tuned by small regulatory RNAs and three sRNAs, namely, CsrB, CsrC and CsrD, have been identified that act as sponges which bind to and inhibit the activity of CsrA. CsrA mutant strains have been observed to have reduced ability to survive drying and form biofilms [56]. Understanding the mechanisms governing the switch in *A. baumannii* can shed light on how to lock the bacterium in an avirulent state to neutralize infection.

## 6. Mechanisms of Antimicrobial Resistance (AMR) of *A. baumannii*

**Figure 3:**
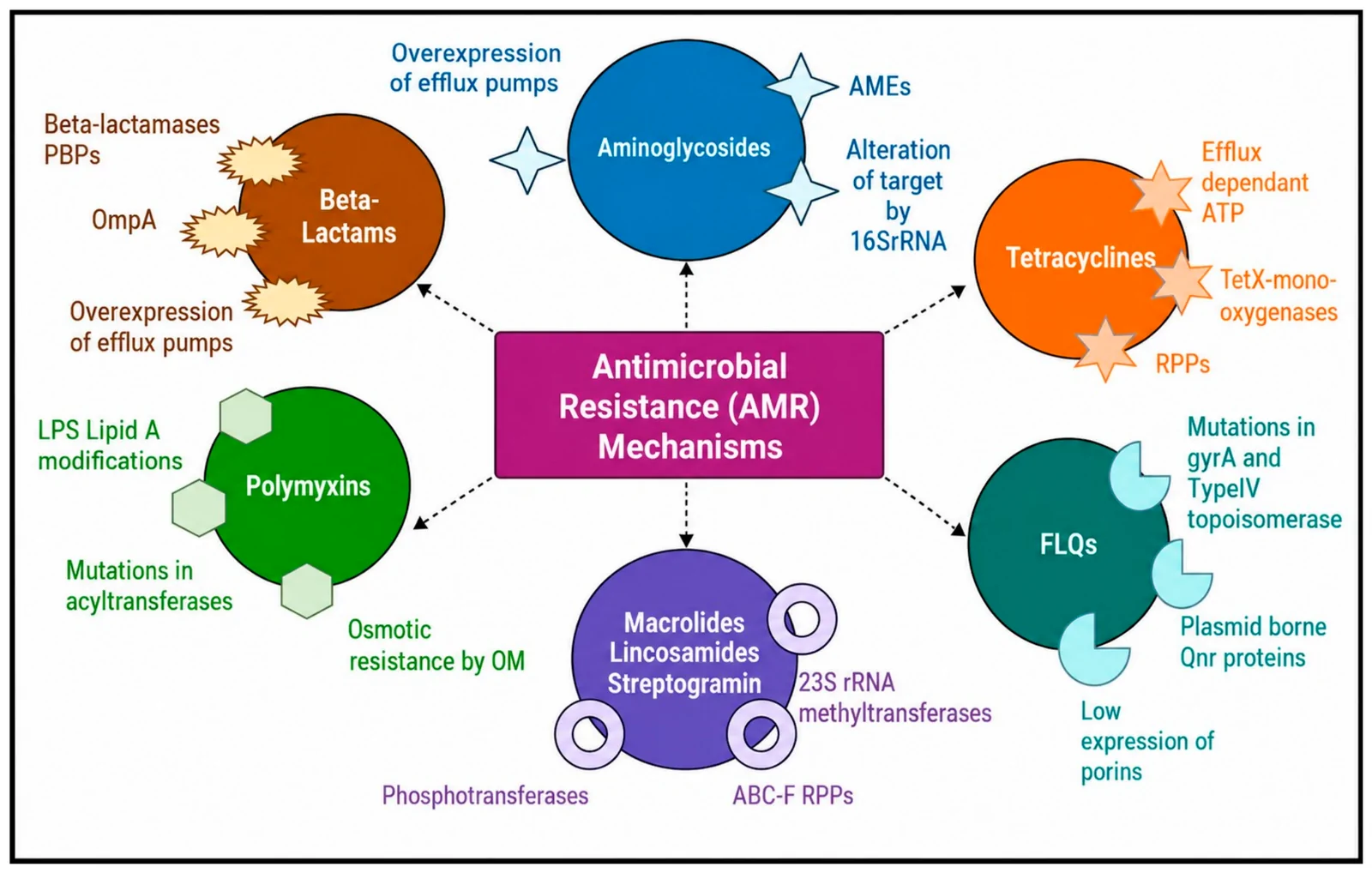
Antimicrobial Resistance Mechanisms exerted by *A. baumannii. PBPSs*: Penicillin-Binding Proteins; OmpA: Outer Membrane Protein A; AMEs: Aminoglycoside-modifying enzymes; 16SrRNA: 16S ribosomal RNA; ATP: Adenosine triphosphate; RPPs: Ribosome protection proteins; gyrA: encodes DNA gyrase enzyme; Qnr proteins: Protect target enzymes from quinolone action; 23s rRNA: 23S ribosomal RNA; ABC: ATP-binding cassette; OM: Outer Membrane; LPS: Lipopolysaccharide. (Source: Image created using references [1,6].).

The mechanisms of antimicrobial resistance (AMR) exhibited by *A. baumannii* are diverse, and understanding these mechanisms helps develop potential solutions to counteract resistance. *A. baumannii* exhibits antimicrobial resistance by various mechanisms, including enzymatic degradation of antibiotics, alteration of target sites, decrease in outer membrane permeability, multidrug efflux pumps, biofilm formation and transfer of resistance determinants via other genetic elements such as plasmids and insertion sequences (Figure 3).

### 6.1. Enzymatic Degradation of Antibiotics

#### 6.1.1. β-Lactamases

β-lactamase production represents the principal enzymatic mechanism driving resistance against advanced-generation cephalosporins, carbapenems and novel siderophore-conjugated β-lactams in *A. baumannii* [33,34]. While newer agents like cefiderocol are engineered to bypass classic resistance pathways via active iron-transport-mediated uptake, the expanded substrate specificity of specific β-lactamases continues to challenge clinical efficacy worldwide [57,58].

Class A extended-spectrum β-lactamases (PER-Type): The enzymes belonging to the PER family, and especially PER-1 poses a severe threat to these new-generation therapies. Structural variations within the active-site cavity of PER-1 allow it to contribute to hydrolysis of advanced oxyimino-acyl side chains, leading to significantly elevated minimum inhibitory concentrations in clinical carbapenem-resistant *A. baumannii* (CRAB) strains [59].

Class C cephalosporinases (ADC Variants): The intrinsic, chromosome-encoded Acinetobacter-derived cephalosporinases (ADCs), such as the ADC-30 variant, exhibit dynamic expression profiles and can be actively induced upon exposure to β-lactam substrates [60]. While basal levels of native class C enzymes do not fully compromise the modern drugs, regulatory mutations or strong upstream insertion sequences drive marked overexpression [34,60]. This excessive enzyme secretion can expand the functional hydrolytic capacity of the cell, enabling enhanced hydrolysis of advanced cephalosporin backbones [60].

Synergy in enzymatic and structural AMR: Robust clinical resistance profiles are rarely isolated to a single enzyme. Rather, they are driven by the multi-layered coproduction of Class A, C and D oxacillinases operating alongside non-enzymatic mechanisms, such as the downregulation of siderophore receptor proteins or altered porin channels, facilitating reduced susceptibility to β-lactam therapy across multidrug-resistant lineages [33,59].

The β-lactamases, their substrate profiles and other enzyme-mediated mechanisms that degrade aminoglycosides and tetracyclines are shown in Table 2 [33,34,60]. An analysis of cefiderocol-non-susceptible isolates from China revealed that these strains were positive for blaPER genes concurrently with blaOXA-23 and blaTEM [58]. Similarly, a New York surveillance study evaluating 34 CRAB isolates with significantly elevated cefiderocol minimum inhibitory concentrations (MICs) demonstrated that they harbored an SHV ESBL; notably, regression analysis revealed no statistical correlation between cefiderocol MICs and the expression of genes encoding *ampC*, blaOXA-51, or the efflux pumps AdeB and AbeM [57]. This strong association with PER-type enzymes is supported by another study reporting that blaPER-1 was ubiquitously present in all cefiderocol-non-susceptible isolates, whereas it was entirely absent in susceptible strains [59]. Crucially, the addition of a potent β-lactamase inhibitor restores the in vitro susceptibility of cefiderocol against these PER-1-producing strains.

#### 6.1.2. Aminoglycoside-Modifying Enzymes

*A. baumannii* employs aminoglycoside-modifying enzymes (AMEs) that chemically modify aminoglycosides, thereby preventing efficient binding of the antibiotics to their ribosomal targets. These AMEs covalently modify either the amino (-NH2) or hydroxyl (-OH) groups of the aminoglycoside molecule. Specifically, aminoglycoside acetyltransferases (AAC) catalyze the acetylation of the -NH2 group, aminoglycoside nucleotidyl transferases (ANT or AAD) mediate modification via O-nucleotidylation, and aminoglycoside phosphotransferases (APH) phosphorylate the -OH group. One of the most prevalent phosphor transferases in *A. baumannii* is APH(3′)-VIa (encoded by the *aphA6* gene). It is well known for conferring resistance to neomycin, kanamycin, amikacin, butirosin and isepamicin. Aminoglycoside nucleotidyl transferases (ANTs) are classified into five distinct groups, with their corresponding genes being located on chromosomes, plasmids or transposons [1,61]. Among clinical isolates, the most common aminoglycoside resistance gene responsible for resistance to kanamycin, tobramycin and gentamicin is *aadB* [34]. Other resistant genes frequently reported in clinical settings include *ant(3″)-Ia*, *aac(3)-Ia*, *aph(3′)-Ia*, *aac(6′)-Ib*, and *aph(3′)-IIb* [33] (Table 2).

### 6.2. Augmented Efflux Pump Mechanisms

In *A. baumannii*, the resistance–nodulation–cell division (RND) family constitutes the major multidrug efflux system contributing to its antimicrobial drug resistance. RND efflux pumps are tripartite complexes composed of an outer membrane factor (OMF), an inner membrane RND transporter and a periplasmic adapter protein (PAP). The PAP and RND transporter form a continuous channel spanning the bacterial cell envelope, thereby facilitating the extrusion of antimicrobial agents from the cell [32]. Several RND efflux systems have been identified in *A. baumannii*, and among these, AdeABC, AdeIJK and AdeFGH are considered clinically significant. The AdeABC efflux system comprises AdeB, the multidrug transporter responsible for substrate extrusion; AdeA, a membrane fusion protein; and AdeC, the outer membrane channel protein. AdeABC confers resistance to a broad range of antimicrobial agents, including aminoglycosides, trimethoprim, chloramphenicol, fluoroquinolones, tetracyclines, macrolides, imipenem and meropenem. Similarly, AdeIJK mediates resistance to chloramphenicol, β-lactams, fluoroquinolones, and tetracyclines, while AdeFGH has been associated with resistance to trimethoprim, tetracycline and tigecycline [1]. Among these systems, AdeABC is the most frequently overexpressed efflux pump in multidrug-resistant *A. baumannii* isolates [62].

The AdeSR two-component regulatory system regulates expression of AdeABC operon, comprising the sensor histidine kinase AdeS and the cytoplasmic response regulator AdeR. Earlier studies have demonstrated that substitution mutations within the autophosphorylation domain of AdeS or the receiver domain of AdeR can lead to constitutive activation and overexpression of the AdeABC efflux pump, thereby increasing antimicrobial resistance levels [63,64,65]. In addition to regulating efflux pump expression, the AdeSR regulon has also been implicated in biofilm formation and virulence [66]. Insertion of ISAba1 into the adeS gene has been demonstrated to increase adeB expression, promoting antibiotic efflux and contributing to tigecycline resistance [67,68].

Other efflux systems have also been implicated in antimicrobial resistance in *A. baumannii*. The AbaQ transporter has been linked to fluoroquinolone resistance, while the EmrAB efflux pump, belonging to the major facilitator superfamily (MFS), has been implicated in reduced susceptibility to colistin [69]. In addition, tetracycline-specific MFS transporters such as TetA and TetB mediate tetracycline efflux. TetA, in interaction with RND efflux systems, may also contribute to tigecycline efflux from the periplasm to the outer membrane [70].

### 6.3. Reduced Outer Membrane Permeability

As a primary barrier to antimicrobial entry, alterations in outer membrane permeability act in close synergy with active efflux systems to drive resistance in *A. baumannii*. While efflux pumps actively export antibiotics from the bacterial cell, outer membrane proteins (OMPs) regulate membrane permeability and thereby influence antibiotic influx. Interaction of OmpA with the peptidoglycan layer contributes to outer membrane stability [71]. The binding of the C-terminal domain of OmpA to the diaminopimelic acid residues of the peptidoglycan layer anchors the outer membrane; disruption of the structural coupling accelerates outer membrane vesicle (OMV) biogenesis. Altered OMP profiles reduce antibiotic influx, whereas OMVs contribute to antimicrobial resistance by sequestering antibiotics and transporting antibiotic inactivating enzymes [72].

Reduced carbapenem susceptibility may also result from loss or structural alteration of the CarO porin, particularly when combined with carbapenemase production and efflux pump overexpression. These structural modifications drastically reduce outer membrane permeability to specific hydrophilic solutes. Along with HMP-AB and OmpW, CarO facilitates the passive transport of β-lactam antibiotics across the membrane, resulting in carbapenem non-susceptibility. Notably, exposure of *Acinetobacter* to sub-inhibitory concentrations of imipenem triggers an adaptive response that significantly reduces CarO expression, severely, limiting antibiotic influx and establishing resistance [73].

This adaptive capability extends to novel iron-conjugated agents. In cefiderocol non-susceptible clinical isolates, reduced expression or transcriptional downregulation of the TonB–ExbB–ExbD energy transduction system is frequently observed [59]. Disruptive genetic mutations, such as transposon insertions or single-nucleotide polymorphisms within *exbD* can disrupt the functionality of this outer-membrane energizing complex. Since cefiderocol targets and exploits active siderophore-mediated iron transport pathways to achieve cellular entry, impairment of the TonB–ExbB–ExbD system effectively cuts off this entry route, decreasing intracellular drug accumulation and driving high-level cefiderocol resistance [59].

### 6.4. Alteration of the Target Site

Alteration of the target site or cellular function is commonly seen in *Acinetobacter* against fluoroquinolones, colistin and ceftazidime–avibactam. The most common mechanism of fluoroquinolone resistance is point mutation in the gyrA, gyrB, and parC genes, which encode the enzymes gyrase and topoisomerase IV. A single mutation in gyrA, inducing an amino acid change from serine to leucine in position 83, reduces the susceptibility to fluoroquinolones. The parC mutation at position 80, from serine to isoleucine, and at 84, from glutamic acid to valine, leads to quinolone resistance [74].

The mechanism by which penicillin-binding protein (PBP) contributes to AMR is through modification of the active site, which reduces the affinity of carbapenem or β-lactam binding. Penicillin-binding protein (PBP) catalyzes the polymerization and transpeptidation of peptidoglycan. Inhibition of PBP by β-lactams impairs cell wall synthesis and leads to cell death. The X-ray crystallography of PBP 2 reveals the transpeptidase domain of *A. baumannii*, which has a zinc binding site close to the catalytic core. Zinc is critical for the stability of PBP, and zinc loss leads to hyper susceptibility to β-lactams [75]. Studies reported imipenem resistance in *Acinetobacter* clones with PBP alteration. PBP3 mutants have been associated with resistance to meropenem, sulbactam and cefiderocol [1].

Methylation of 16S rRNA of the 30S ribosomal subunit changes the target site for aminoglycoside binding. The armA gene encodes methyltransferases, which confer high-level resistance to gentamicin. Class 1 integrons transfer them and are commonly seen in CRAB [61]. The other reported genes are rmtB, rmtB1 and rmtE, which confer resistance to all aminoglycosides [1,61,76]. Colistin resistance occurs through complete loss of lipopolysaccharide (LPS) due to inactivation of the lpxA, lpxC, or lpxD genes. Loss of the target prevents polymyxins, including colistin, from binding to LPS, rendering them ineffective.

Mutations in the *ftsI* gene, particularly the I236N and H370Y substitutions within the transpeptidase domain of penicillin-binding protein 3 (PBP3), induce structural alterations that contribute to elevated cefiderocol MICs. Additionally, a missense mutation in *mrcB* (R363C), which encodes penicillin-binding protein 1b (PBP1b), has been concurrently implicated in reduced cefiderocol susceptibility [77,78].

Beyond target-site modifications, alterations in genes governing iron transport systems further drive resistance. Mutations and reduced expression of the siderophore receptor genes *piuA* and *pirA* have been widely identified among cefiderocol-non-susceptible strains. Furthermore, the downregulated expression of crucial iron transport-related genes, including *fiu*, *feoA*, *feoB*, *tonB*, and *exbB* in isolates exhibiting elevated cefiderocol MICs firmly establish that impaired iron uptake mechanisms play a pivotal role in mediating resistance [59].

### 6.5. Other Mechanisms

#### 6.5.1. Insertion Sequence (IS) in Acinetobacter

About 30 types of IS in *Acinetobacter* have been reported, and the most prevalent is ISAba1. ISAba1 is responsible for the transfer and expression of increased carbapenem resistance. Overexpression of the ISAba1 promoter drives transcription of AdeIJK efflux pump genes [79], which confers broader resistance to β-lactams, tetracyclines, aminoglycosides and tigecycline. ISAba825, ISAba125, ISAba10 and ISAba27 insertions disrupt the CarO gene, which encodes OMP of *Acinetobacter.* ISAba1, ISAba125 and ISAba27 disrupt the adeN repressor of the AdeIJK efflux pumps, leading to resistance to multiple antibiotics [33]. An ISAba11 insertion inactivates IpxA or IpxC, leading to a loss of LPS production and enhanced colistin resistance. ISAba1 transposition upstream of the eptA gene increases its expression, thereby reducing the negative charge on the cell membrane, and reducing the affinity for colistin [80].

#### 6.5.2. Integrons

To date, five classes of integrons are associated with resistance in *Acinetobacter* [81]. The most common types of transposable integrons are class I, derived from Tn402, class II, derived from Tn7 and class III. These three integrons share genetic identity and confer resistance to aminoglycosides, quinolones, cephalosporins, sulfonamides, tetracyclines, and chloramphenicol. Type 1 integrons are responsible for aminoglycoside resistance (aadB, aacA4, aacC1) and β-lactam resistance (IMP, VIM, SIM) [82].

#### 6.5.3. Plasmid-Mediated Resistance and Resistance Islands

The plasmid typing based on the replication initiation gene (rep) of *Acinetobacter* revealed three types, R1, R3, RP, and a rep-less group. The R3, RP, and rep-less groups are responsible for the transfer of antibiotic resistance [83]. R3 plasmids constitute a diverse group, have a wide geographical distribution and are also reported in major global clones such as GC1 and GC2. R3 plasmids are associated with blaOXA-58, blaOXA-72 and blaOXA-24, responsible for β-lactam resistance, tet39 (tetracycline resistance), sul2 (sulfonamide resistance), and the mcr gene (colistin resistance) [83,84]. RP-T1 carries blaOXA-23 and/or aphA6; these plasmids are reported in major clones such as GC1, GC2, ST 10, ST 15, ST 25, ST 79 and ST 622 [83]. The small plasmids pRAY and its variants are known to carry the aadB gene, which confers resistance to tobramycin, gentamicin, and kanamycin, and have been reported in ST1, ST82, ST2, ST25, and ST85.

The MPFF conjugative plasmid harbors resistance genes msr-mph (E) conferring macrolide resistance, blaPER-7 conferring ESBL resistance and arm A genes encoding aminoglycoside resistance. MPFT conjugative plasmids are known to harbor blaNDM, which rapidly disseminates [33].

The AbaR resistant island family is commonly seen in GC1 and GC2 clones. GC1 and GC2 lineages contain resistance islands with a Tn6019 backbone in the comM gene; the central region of the transposon has antibiotic resistance genes. AbaR4 resistant islands have been identified in chromosomes and on conjugative plasmids and are responsible for carbapenem resistance. In GC2 lineage, ABGRI 1–5, located at distinct chromosomal sites, have a distinct transposon backbone structure and are responsible for resistance to tetracyclines, aminoglycosides, extended-spectrum cephalosporins (blaTEM) and carbapenems (OXA-23) [85].

### 6.6. The Interplay Between Virulence, Biofilm Architecture and Resistance Phenotypes

While the mechanisms of AMR detailed above are generally conceptualized as independent evolutionary traits, in *A. baumannii,* they form a highly integrated regulatory and functional network with virulence determinants [86]. A primary axis of this interplay is mediated by quorum-sensing systems, particularly the *abaR*/*abaI* locus. QS influences the expression of numerous virulence-associated genes involved in biofilm development and host adaptation [87]. Within the biofilm matrix, restricted antibiotic diffusion acts synergistically with the overexpression of multidrug efflux pumps, including AdeABC, to enhance antimicrobial tolerance in many clinical isolates [63,87]. This convergence significantly elevates bacterial tolerance to antimicrobial agents, protecting the pathogen against clinically relevant agents, including carbapenems and aminoglycosides [63,87]. Furthermore, this co-regulation entails distinct metabolic and fitness costs [86]. The acquisition of high-level resistance, such as target-site mutations in *gyrA*/*parC* or the energetic burden associated with maintaining carbapenemase determinants, may result in a temporary reduction in growth rate or initial virulence in vivo [86,88]. However, these fitness deficits may be mitigated through compensatory mutations and the emergence of metabolically dormant persister cells within the biofilm matrix [87]. Consequently, the regulatory crosstalk between density-dependent signaling, biofilm-mediated physical protection and AMR gene expression forms a self-reinforcing survival loop that promotes long-term persistence without permanently sacrificing pathogenicity [63,86].

## 7. Treatment Strategies—Re-Equipping the Armamentarium

### 7.1. Antibiotic Choices

The emergence of MDR and carbapenem-resistant *A. baumannii* (CRAB) strains in clinical settings limits the selection of effective antibiotics for treating these infections in humans. Despite the availability of advanced antimicrobial therapies, infections caused by MDR *A. baumannii* continue to be associated with substantial morbidity and mortality. An epidemiological study has reported intensive care unit (ICU) mortality rates of 20% among patients infected with MDR *A. baumannii*, highlighting the urgent need for improved therapeutic strategies and strengthened antibiotic stewardship programs [89]. Higher levels of resistance to other antibiotics among CRAB further complicate the antibiotic therapy. A study reported that CRAB isolates exhibit more than 50% resistance to gentamicin, piperacillin-tazobactam, amikacin, and cefepime, further complicating antibiotic selection and limiting available treatment options [90,91]. The increasing prevalence of CRAB has necessitated the use of last-line antibiotics, particularly polymyxins such as colistin. Colistin remains one of the most active agents against MDR *A. baumannii*. Clinical surveillance studies have shown that although many isolates exhibit high levels of resistance to β-lactams, fluoroquinolones, aminoglycosides, and carbapenems, they often remain susceptible to colistin [92]. Similarly, surveillance data have reported multiple antibiotic resistance indices ranging from 0.64 to 0.91 among MDR *A. baumannii* isolates. Yet, these strains remain largely susceptible to colistin, reinforcing its continued importance as a last-resort therapeutic option in intensive care settings [93].

In addition to polymyxins, tigecycline has emerged as a highly active therapeutic agent against MDR *A. baumannii.* A study showed that 58.7% of clinical isolates were susceptible to tigecycline, indicating moderate but clinically relevant activity against drug-resistant strains [92]. Furthermore, a surveillance study reported no resistance to tigecycline and colistin among tested isolates, further supporting their potential as treatment options for difficult infections [91].

Combination antibiotic therapy is often used for severe CRAB infections to enhance treatment efficacy. Antibiotics such as ampicillin–sulbactam, tigecycline, and colistin are often used alone or in combination, depending on the isolate’s susceptibility profile. Notably, susceptibility analyses have shown relatively low rates of resistance to ampicillin–sulbactam and polymyxin B, suggesting that these agents may retain activity against MDR *A. baumannii* isolates [94]. However, it is critical to note that the intrinsic antibacterial efficacy of ampicillin–sulbactam and sulbactam–durlobactam against *A. baumannii* is mediated by sulbactam itself through the direct targeting and inhibition of PBP1 and PBP3 [95,96]. In these regimens, the companion β-lactam (ampicillin) exerts no significant antimicrobial activity against the pathogen, serving historically as a vehicle because standalone sulbactam formulations were not available [97].

In a randomized clinical trial involving 423 patients, the 28-day mortality was 43% with colistin monotherapy and 37% with colistin–meropenem combination therapy, showing no significant clinical survival benefit from adding meropenem. Microbiological cure rates were similar between the two groups (65% with monotherapy and 60% with combination therapy). Acute kidney injury occurred in about 49–52% of patients, indicating substantial nephrotoxicity associated with colistin treatment [98]. Another important option is sulbactam, which has intrinsic activity against *A. baumannii*. A meta-analysis of 18 studies and 1835 patients with MDR/XDR infections found that high-dose sulbactam (≥6 g/day), in combination with tigecycline or levofloxacin, was associated with greater clinical improvement and cure rates [99]. A case study demonstrated that an 83-year-old female patient with CRAB bacteremia showed no clinical improvement with conventional therapy consisting of ampicillin–sulbactam and colistin. However, the addition of ceftazidime–avibactam (940 mg every 12 h) to ampicillin–sulbactam resulted in rapid clinical recovery, including resolution of fever and discontinuation of vasopressor and ventilatory support. These findings suggest that the sulbactam–avibactam combination may serve as a promising treatment option for severe CRAB infections, particularly when standard therapies fail or cause toxicity [100].

In recent years, several novel antimicrobial agents have expanded the treatment armamentarium against MDR *A. baumannii*, particularly cefiderocol, sulbactam–durlobactam, and eravacycline. Among these, sulbactam–durlobactam has emerged as an advanced therapeutic option for infections caused by CRAB. Sulbactam exhibits intrinsic antibacterial action against *A. baumannii* through its binding to penicillin-binding proteins, while durlobactam protects it from hydrolysis by class A, C, and D β-lactamases. Its approval for hospital-acquired and ventilator-associated pneumonia caused by the *A. baumannii–calcoaceticus* complex is supported by clinical studies that show better treatment results and less nephrotoxicity [101,102].

Cefiderocol is a novel siderophore cephalosporin that overcomes traditional antibiotic resistance mechanisms by entering cells via bacterial iron-transport systems. Due to its novel mechanism of action, cefiderocol has demonstrated activity against CRAB, including in combination regimens. However, the emergence of cefiderocol-resistant isolates highlights the need to select antibiotics based on the susceptibility pattern and to continue surveillance [102,103].

A synthetic fluorocycline antibiotic, eravacycline, has also recently been considered for treating infections caused by MDR *A. baumannii*. Structural modifications allow eravacycline to evade common tetracycline resistance mechanisms, including efflux pumps and ribosomal protection proteins. In vitro studies revealed potent activity of eravacycline against CRAB isolates, often exhibiting lower MIC than tigecycline [104]. More recently, successful treatment of cefiderocol-resistant *A. baumannii* bloodstream infection with eravacycline monotherapy has been reported, highlighting its potential as a salvage therapeutic option in selected patients with limited treatment regimens [105].

### 7.2. Non-Antibiotic Therapeutic Approaches

#### 7.2.1. Bacteriophage Therapy

Bacteriophages are abundant in the environment and exhibit high host specificity toward bacteria. This specificity is largely determined by recognition of bacterial cell-surface receptors, including outer membrane porins (OmpA, OmpC, and LamB), lipopolysaccharides, capsular polysaccharides, and lipoproteins. Because of this receptor-mediated specificity, bacteriophages are considered highly targeted antibacterial therapeutic agents [98,99]. Bacteriophages selectively lyse susceptible bacterial cells and generally exhibit limited direct toxicity to mammalian cells. In recent years, phages have gained attention as potential therapeutic agents against MDR bacterial infections, with several clinical cases demonstrating successful outcomes [106,107,108]. Table 3 lists the bacteriophages isolated against *A. baumannii.* For example, bacteriophage cocktails were administered intravenously and percutaneously to a 68-year-old diabetic patient with necrotizing pancreatitis caused by MDR *A. baumannii*. The patient did not respond to conventional antibiotic therapy, but phage treatment effectively cleared the infection [109]. The study also identified that phage isolates are susceptible to minocycline, highlighting that phages selected for therapy should also be evaluated for their interactions with antibiotics.

In another case study, bacteriophages were delivered through inhalation to treat respiratory infections caused by extensively drug-resistant (XDR) *A. baumannii.* During the initial phase of treatment, the abundance of phage in sputum samples increased, while the bacterial load decreased, suggesting active phage replication and bacterial lysis in the respiratory tract. However, in the last phase, bacterial levels remained relatively stable despite continued phage administration, indicating the possible emergence of phage tolerance or resistance. Additionally, 16S rRNA sequencing revealed a remarkable shift in gut microbiota composition during treatment, including an increase in Proteobacteria and fluctuations in other bacterial genera. Although the patient also received concurrent antibiotic therapy, these findings suggest that phage therapy may influence both pathogen burden and host microbial composition during infection treatment [108].

The bacteriophage vB_AbaS_SA1, isolated from hospital sewage, has shown strong antibacterial activity against MDR *A. baumannii* isolates. The phage produced clear plaques and displayed Sipho virus morphology. Host range analysis indicated that SA1 could infect approximately one-third of MDR *A. baumannii* clinical isolates, and showed no activity against other Gram-negative bacteria. Combinations of phage SA1 with antibiotics significantly enhanced antibacterial activity, reducing the minimum inhibitory concentrations of meropenem, ampicillin–sulbactam, and colistin by at least twofold across all tested isolates. Time-kill assays further confirmed strong phage–antibiotic synergistic activity, demonstrating enhanced bacterial suppression compared with either treatment alone [107].

A phage cocktail composed of nine lytic bacteriophages, administered intravenously and directly into infected abscess cavities, successfully treated MDR *A. baumannii* infection in a patient with necrotising pancreatitis, resulting in complete clinical recovery after the failure of conventional antibiotic therapy [109]. This study reported the use of bacteriophage therapy to treat a severe MDR *A. baumannii* infection at a craniectomy site in a 77-year-old patient. Five lytic phages active against the patient’s isolate were identified from a phage library, and the most virulent phage was provided intravenously at a dose of approximately 2 × 10^10^ PFU/mL every two hours for eight days. Although local wound healing was observed, the patient died because of the severity of the underlying trauma. The study highlights both the potential safety of phage therapy and the need for controlled clinical trials to determine optimal dosing, routes of administration and therapeutic efficacy [126].

In a pre-clinical murine bacteremia model study, Gordillo Altamirano et al. demonstrated that bacteriophage øFG02 significantly reduced the burden of MDR *A. baumannii*, and its combination with ceftazidime was more effective than either treatment alone. Phage therapy drove the emergence of phage-resistant mutants in 96% of treated animals; however, these mutants lost their capsule and became more susceptible to ceftazidime. The combination therapy achieved sustained bacterial suppression and reduced bacterial loads by up to 3.7 logs compared with untreated controls. The study highlights the potential of phage–antibiotic combination therapy as a promising strategy against MDR *A. baumannii* [127]. In another study, in vitro experiments showed that lytic phages combined with ceftazidime reduced bacterial load by more than 85% and helped overcome phage resistance observed during phage-only treatment [112].

#### 7.2.2. Antimicrobial Peptides (AMPs)

The emergence of MDR *A. baumannii* has limited the effectiveness of conventional antibiotics and has prompted researchers to seek alternative therapeutic strategies [128]. Antimicrobial peptides (AMPs) have been investigated as potential alternatives to conventional antibiotics due to their broad-spectrum antibacterial potential. AMPs are short peptides composed of five to one hundred amino acids, primarily involved in the innate immune system of many organisms against invasive pathogens. AMPs mostly interact with bacterial membranes via electrostatic attraction between their cationic residues and the negatively charged membrane surface, in contrast to conventional antibiotics, which typically target specific metabolic pathways. This interaction compromises membrane integrity, causes leakage of intracellular contents, and ultimately results in bacterial cell death [129].

A designed peptide, Ω76, exhibited antibacterial activity against MDR *A. baumannii* strains. In particular, Ω76 was shown to rapidly compromise the integrity of bacterial membranes in experimental models by adopting an α-helical conformation, leading to cytoplasmic leakage and bacterial death. Ω76 therapy markedly increased lifespan in animal infection models without causing any notable harm, demonstrating the therapeutic promise of peptide-based antibiotics [130]. Recent developments in peptide engineering have created synthetic or modified AMPs with improved stability, reduced toxicity and enhanced antimicrobial activity. For instance, de novo-designed peptides such as PepD2 and its derivatives have shown potent bactericidal activity against *A. baumannii*, with minimum bactericidal concentrations of 4 μg/mL. Furthermore, incorporation of D-amino acids has been reported to enhance peptide stability and extend their half-life in plasma, thereby increasing their potential for therapeutic applications [131].

AMPs offer advantages over conventional antibiotics, including the ability to penetrate biofilms or inhibit their formation, thereby addressing a major mechanism underlying chronic and recurrent *A. baumannii* infections. The antimicrobial peptide Cec4 has been shown to disrupt bacterial membranes, enter bacterial cells and significantly reduce biofilm formation while exhibiting minimal cytotoxicity toward host cells [132]. Similarly, chimeric antimicrobial peptides have been found to show potent antibacterial and antibiofilm activities against MDR *A. baumannii* isolates. These peptides demonstrated MICs ranging from approximately 3.12 to 12.5 µM against MDR strains and exhibited synergistic effects when combined with conventional antibiotics such as ciprofloxacin or cefotaxime. Such synergistic interactions may enhance therapeutic effectiveness while reducing the required antibiotic dosages [131].

A research group developed antimicrobial peptide databases and computational pipelines to identify novel AMP candidates targeting *A. baumannii*. Resources such as AbAMPdb (https://abampdb.mgbio.tech/accessed on 1 October 2024) provide curated datasets of experimentally validated and synthetic AMPs, enabling researchers to screen peptides for interactions with bacterial virulence factors and resistance proteins [133]. A recently designed peptide (T2-02) using computational approaches demonstrated strong activity against CRAB isolates, with MICs ranging from 8 to 16 µg/mL and low cytotoxicity toward mammalian cells. The incorporation of liposomal nano delivery systems further enhanced the peptide’s antimicrobial activity and stability, supporting advanced drug-delivery approaches and potentially improving AMP-based therapies [134]. Along with these promising properties, peptide stability and susceptibility to proteolytic degradation, potential cytotoxicity, and high production costs remain challenges to the clinical use of AMPs.

Although AMPs have demonstrated promising antibacterial, antibiofilm and synergistic activities against MDR *A. baumannii*, the majority of the current evidence is derived from in vitro studies, computational analyses and animal infection models. Consequently, most AMP-based therapies remain at the preclinical stage of development, and only limited clinical data are currently available to support their routine therapeutic use. Challenges regarding peptide stability, susceptibility to proteolytic degradation, potential cytotoxicity, manufacturing costs and large-scale production must be addressed before widespread clinical translation can be achieved.

#### 7.2.3. Vaccine-Based Therapy

Vaccination is a promising strategy because it can stimulate long-lasting immunity, reduce bacterial transmission and potentially limit the emergence of AMR in bacteria. Whole-cell and chemically inactivated bacterial preparations have been investigated for vaccination against *A. baumannii*. These vaccines simultaneously expose the immune system to a wide range of bacterial antigens, which may generate broad protective immunity. Formalin-inactivated whole-cell vaccines have demonstrated encouraging results in animal models, where immunized mice exhibited reduced bacterial loads and improved survival following lethal challenge with virulent strains. These vaccines can stimulate both humoral and cellular immune responses due to the presence of multiple antigenic components. However, the presence of endotoxins such as lipopolysaccharides (LPSs) may induce inflammatory responses and toxicity, thereby limiting their development for clinical use [91,135].

Subunit vaccines have been considered as a safer alternative to whole-cell vaccines. These vaccines contain purified antigenic components of the pathogen rather than the entire organism, thereby reducing the risk of adverse reactions. In *A. baumannii*, several OMPs have been identified as promising vaccine targets due to their surface exposure and involvement in bacterial virulence. Outer membrane protein A (OmpA) is one of the extensively studied antigens that is highly conserved among different *A. baumannii* strains and is responsible for adhesion, invasion, biofilm formation and host immune modulation. Immunization with recombinant OmpA induces strong antibody responses and protective immunity in murine infection models. In addition to OmpA, other protein candidates, namely Omp22, Omp33–36, OmpW, Ata, and fimbrial proteins such as CsuA/B and FimA, have also demonstrated immunogenic potential. Subunit vaccines often exhibit relatively weak immunogenicity and rapid degradation in vivo. Furthermore, the use of adjuvants or delivery systems is required to enhance antigen stability and stimulate stronger immune responses [91,136].

Outer membrane vesicles (OMVs) are another promising vaccine platform in Gram-negative bacteria. OMVs are naturally secreted from the bacterial outer membrane and contain various immunogenic components such as outer membrane proteins, lipopolysaccharides and periplasmic proteins. Because OMVs mimic the structural composition of the bacterial surface, they can effectively stimulate both innate and adaptive immune responses. Studies have shown that OMV-based vaccines can elicit strong antibody responses and protect against *A. baumannii* infections in animal models. Moreover, OMVs present multiple antigens simultaneously, which may increase the likelihood of cross-protection against diverse clinical isolates. However, the presence of endotoxin within OMVs requires careful detoxification or genetic modification to minimize inflammatory toxicity [91,136].

Multi-epitope vaccines combine multiple B-cell and T-cell epitopes from different antigens to confer broader immune protection. Using bioinformatic tools, conserved immunogenic epitopes were identified in OMP and other virulence factors of *A. baumannii*. These epitopes can be assembled into synthetic vaccine constructs designed to stimulate both humoral and cellular immune responses. Recent computational studies have predicted multi-epitope vaccine candidates that bind strongly to host immune receptors such as Toll-like receptors (TLR2 and TLR4). Immune simulations suggest that such constructs may induce strong antibody production, cytokine secretion and T-cell activation [137,138,139].

Advanced genomics and computational methods can also predict antigenicity, allergenicity, and interactions with immune receptors prior to laboratory validation, thereby reducing the time and cost of vaccine discovery. Through these approaches, several promising vaccine targets have been identified, including proteins involved in membrane transport, adhesion and bacterial virulence. Although these strategies have produced encouraging results in preclinical studies, further experimental validation and clinical evaluation will be necessary to translate these candidates into effective vaccines. Currently, no vaccine candidate against *A. baumannii* has yet progressed to advanced clinical evaluation, reflecting the significant challenges involved in developing an effective vaccine against this pathogen [91,137,140].

#### 7.2.4. Monoclonal-Antibody-Mediated Therapy

Monoclonal antibody (mAb)-mediated therapy is a promising strategy to combat infections caused by *A. baumannii*. mAbs recognize specific bacterial surface antigens and enhance host immune responses. These antibodies can promote bacterial clearance through opsonization, complement-mediated killing, neutralization of virulence factors and enhancement of phagocytosis by immune cells. Studies investigating antibody-based therapies have demonstrated that passive immunization can significantly reduce bacterial load and improve survival in experimental infection models [141,142].

The capsule of *A. baumannii* plays an essential role in virulence by protecting the bacterium from host immune defences and facilitating persistence in host tissues. It has been demonstrated that mAbs targeting capsular polysaccharides enhance complement deposition and opsonophagocytic destruction of *A. baumannii*. The potential of capsule-targeted immunotherapy has been demonstrated in animal infection models, where passive delivery of anti-capsular antibodies significantly increased survival rates and reduced bacterial dissemination [143,144].

mAbs targeting outer membrane protein A (OmpA) specifically bind to its surface-exposed epitopes and facilitate immune-mediated clearance of the pathogen. Anti-OmpA mAbs enhance macrophage-mediated killing of *A. baumannii* strains such as AB307.30 by promoting opsonophagocytosis. However, the effectiveness of these antibodies may vary among clinical isolates due to structural barriers such as capsular polysaccharides that mask OmpA epitopes [145]. mAbs can also recognize surface carbohydrate antigens, including capsular polysaccharides and LPS, enabling specific binding to bacterial cells. For example, monoclonal antibody MAb10 binds carbohydrate motifs containing pseudaminic acid present in *A. baumannii* surface polysaccharides. The binding of such antibodies promotes immune clearance by enhancing macrophage-mediated opsonophagocytosis and bacterial uptake. Experimental studies have shown that monoclonal antibodies against these surface polysaccharides can display strong in vitro binding and protective activity against *A. baumannii* infections [146]. The antigenic diversity of capsular polysaccharides among different *A. baumannii* strains may limit the coverage of individual antibodies, and the cost of antibody production may also present challenges.

#### 7.2.5. Antimicrobial Photodynamic Treatment

Antimicrobial photodynamic therapy (aPDT) is a non-antibiotic technique that kills pathogens in humans using a non-toxic photosensitizer. The photosensitizer is activated by light of a specific wavelength in the presence of oxygen. Upon photoactivation, the photosensitizer produces reactive oxygen species (ROS), including singlet oxygen, superoxide, and hydroxyl radicals. These ROS cause oxidative damage to the lipids, proteins, nucleic acids and bacterial cell membranes. aPDT may delay the emergence of resistance in bacterial pathogens compared to conventional antibiotic treatment [147].

Several studies have demonstrated the efficacy of aPDT against planktonic and biofilm forms of *A. baumannii*. Methylene blue-mediated aPDT significantly reduced viable bacterial counts in both reference and clinical MDR strains. In planktonic cultures, reductions ranged from approximately 2 log_10_ CFU to complete bacterial inhibition, while biofilm-associated cells showed reductions of up to 3.9 log_10_ CFU following treatment [148]. In addition to direct bacterial killing, aPDT may enhance the activity of conventional antibiotics. Sub-lethal photodynamic inactivation has been shown to increase the susceptibility of XDR *A. baumannii* to several antibiotics. The mechanism appears to involve ROS-mediated damage to the bacterial envelope and cellular structures, thereby increasing antibiotic penetration and disrupting resistance mechanisms [147].

Riboflavin- and chlorophyllin-based aPDT systems have demonstrated the ability to generate intracellular ROS and effectively reduce *A. baumannii* viability, even within biofilms. The ability of aPDT to disrupt biofilm structures and damage extracellular polymeric matrices represents an important therapeutic advantage [149].

Although aPDT has shown promising results, several limitations must be overcome before aPDT can be widely used to treat *A. baumannii* infections. The therapeutic efficacy of aPDT is influenced by multiple factors, including the type of photosensitizer used, the wavelength and dose of light, oxygen availability and the physiological state of bacterial cells. Further well-designed clinical studies are needed to evaluate the long-term safety and therapeutic efficacy of aPDT.

### 7.3. Translational Barriers, Safety Profiles and Clinical Evidence Deficits

While the alternative therapeutic pipeline appears promising in preclinical research, significant hurdles to clinical integration persist due to distinct translational bottlenecks.

Bacteriophage therapy: Despite impressive lytic efficacy demonstrated in murine studies, human applications may be rapidly cleared by the host mononuclear phagocyte system. Additionally, clinical trials show that *A. baumannii* can rapidly adapt and develop phage resistance through mutations in surface receptors. Standardized regulatory frameworks for large-scale manufacturing of dynamic, multi-phage cocktails remain challenging to establish.

Antimicrobial peptides (AMPs): While a few peptides like Cec4 and Ω76 show remarkable ability to destroy target membranes, their translation could be hindered by the high cost involved in chemical synthesis and production. Furthermore, native linear peptides have short metabolic half-lives due to proteolytic degradation in human serum and carry a risk of systemic toxicity.

Vaccine interventions: In murine studies, whole-cell and multi-antigen-inactivated vaccine candidates have shown broad protective immunogenicity. However, the residual presence of highly endotoxic lipid-A regions in outer membrane fractions poses significant regulatory concerns about the safety of inflammatory responses in human clinical trials. In the future, large-scale, multicenter randomized controlled clinical trials are necessary to establish clear safety guidelines and functional efficacy in humans.

## 8. Control Strategies for *A. baumannii* Infections

*A. baumannii is one* of the most important pathogens causing hospital-acquired infections, particularly in intensive care units (ICUs), burn wards, and immunocompromised patients. *A. baumannii* can survive for prolonged periods on dry surfaces, medical devices and hospital environments. Biofilm formation enhances bacterial resistance to disinfectants and desiccation. The acquisition of MDR and virulence characteristics is a major contributing factor to treatment failure and higher morbidity and mortality associated with *A. baumannii* infections. Owing to the lack of effective treatment options, infection prevention and control (IPC) strategies remain essential to mitigate the burden of *A. baumannii* infections. Regular IPC training programs to increase awareness among healthcare workers and patients in hospital settings are needed to prevent transmission within the facilities. Standard precautions such as strict hand hygiene, use of personal protective equipment, and environmental decontamination with disinfectants are crucial components [150,151].

Patients infected with MDR *A. baumannii* should be isolated to limit contact with other patients, with dedicated medical equipment, appropriate glove use and other personal protective equipment (PPE). Furthermore, proper laundering of bedclothes, protective clothing, and aprons worn by healthcare personnel might significantly reduce the risk of hospital-acquired infections. Restricting the movement of healthcare personnel between colonized or infected patients and other wards can reduce interdepartmental transmission, particularly in ICUs and burn units. Active surveillance plays an important role in early detection and containment of *A. baumannii* outbreaks. Data on local pathogen prevalence and AMR patterns should be monitored and maintained at the hospital level. Systematic sampling of patient care areas and equipment during suspected outbreaks can help identify sources of infection.

A study described the infection control measures taken to control the *A. baumannii* outbreak in burn units. Outbreak investigation must be performed using the hospital’s bacterial pathogen prevalence data, often by swabbing the dressing room, transport trolley, patients’ beds, and other accessible sites, such as water taps. Strict adherence to glove use and hand hygiene protocols by healthcare professionals will significantly mitigate nosocomial transmission and control pathogen spread within the healthcare facility [152,153]. Seventy per cent ethanol, povidone-iodine and chlorhexidine gluconate are effective agents for hand-cleaning procedures [154].

The control of *A. baumannii* infections requires an integrated strategy encompassing environmental hygiene, active surveillance and antimicrobial stewardship. A sustained institutional commitment and adherence to evidence-based IPC guidelines are critical to reducing transmission and improving patient outcomes [155,156].

## 9. Future Directions and Challenges Ahead

Despite significant strides in understanding the virulence and resistance mechanisms of *A. baumannii*, several challenges remain in translating this knowledge into real-world clinical benefit. Developing point-of-care diagnostic tools for quicker, more accurate detection of *A. baumannii*, along with the ability to differentiate infection from colonization and to profile antimicrobial resistance genes, would be a priority. The pathogen’s survival abilities and emerging antimicrobial resistance continue to pose challenges in clinical settings. While the therapeutic pipeline of newer strategies appears promising, several challenges remain. While phage therapy faces hurdles such as the need for new regulatory frameworks, standardised production, and measures to counter potential phage resistance, antimicrobial peptides must overcome issues of stability, toxicity, and large-scale manufacturing costs. Future research should focus on a multi-omics approach to target multiple virulence mechanisms of *A. baumannii* simultaneously, thereby effectively combatting the emergence of resistance. Effective prevention strategies and infection control measures are equally important in the war against this wily pathogen, especially in low- and middle-income countries with a higher burden. With the advent of new technologies such as artificial intelligence, it is imperative to integrate them into genomic surveillance to enable real-time tracking of emerging clones and prediction of treatment responses. This, in turn, calls for global coordination, sustained investments, and large-scale clinical trials to alleviate the longstanding global threat posed by *A. baumannii*.

## 10. Conclusions

*A. baumannii* has evolved from an obscure environmental bacterium to one of the most critical drug-resistant clinical pathogens in healthcare settings, leading to significant morbidity and mortality. With an armamentarium of virulence factors at its disposal, it is well-equipped to withstand antimicrobial and environmental pressures. The pathogen has left clinicians worldwide with very limited therapeutic options, and the scientific fraternity is actively investigating alternative therapeutic strategies to overcome this global challenge. Managing this threat would require a multi-pronged approach, including accelerated basic science research to better understand virulence and resistance mechanisms, effective infection control, enhanced global surveillance, antimicrobial stewardship, and the development and evaluation of newer therapeutic options. This review summarizes the current understanding of *A. baumannii*’s virulence and resistance mechanisms, and discusses the newer therapeutic pipeline. Global efforts and coordinated funding support are urgently needed for effectively combating this pathogen.

## Figures and Tables

**Table 1:** Quorum sensing components and their functions and virulence mechanisms.

S. NO.	QS Component/Gene	Type	Function in Quorum Sensing	Role in Virulence	Key References
1.	AbaI	Autoinducer synthase (LuxI homolog)	Synthesizes N-acyl homoserine lactone (AHL) signaling molecules	Regulates biofilm formation, motility, virulence gene expression	[45,47]
2.	AbaR	Transcriptional regulator (LuxR homolog)	Binds AHL and activates QS-dependent genes	Controls genes for adhesion, surface motility, and pathogenicity	[45]
3.	AbaM	QS regulatory modulator	Modulates expression of abaI/abaR system	Influences biofilm formation and virulence regulation	[48]
4.	AHL molecules (e.g., OHC12-HSL)	Signaling molecules	Diffuse across membrane; accumulate at high cell density	Trigger coordinated expression of virulence genes	[47]
5.	Outer membrane proteins (OMPs)	Membrane proteins	Some OMP genes under QS influence	Contribute to adhesion and immune evasion	[49]
6.	Efflux pumps (e.g., AdeABC)	Transport proteins	QS may regulate stress response pathways affecting efflux	Contribute to antibiotic tolerance in biofilms	[44]

**Table 2:** Classification of enzymes that inactivate antibiotic groups, their substrate profile and the genes responsible for encoding the enzymes.

Enzymes	Resistance Mediated by	Genes Responsible	Substrate Profile
Class A β-lactamases	Serine-dependent, extended-spectrum activity is caused by point mutations	blaSCO1, blaTEM-92, blaSHV, blaGES-11, blaGES-14, blaPER-1, blaPER-7 &blaVEB-1	Penicillin, extended spectrum cephalosporins including Aztreonam & carbapenems
Class B β-lactamases	Encoded by mobile genetic elements such as plasmids & integrons and the enzyme requires zinc for catalysis	blaVIM-1, VIM-2, VIM-3, VIM-4, VIM-11, IMP-1, IMP-2, IMP-4, IMP-5, IMP-9, IMP-10, SIM-1 & NDM-1	Hydrolysis of all β-lactams, including carbapenems, but not monobactams.
Class C β-lactamases	Serine-dependent; chromosomally encoded cephalosporinase. Overexpression can be induced by the insertion of ISAba1 & ISAba125 upstream of blaADC	*ampC*/Acinetobacter derived cephalosporinase ADC	Resistant to cephalosporin, carbapenems and sulbactam
Class D β-lactamases	Serine-dependent; chromosomal/plasmid-mediated enzymes.	Oxacillinases/carbapenem hydrolyzing class D β-lactamases (blaOXA-10, blaOXA-23, blaOXA-24, blaOXA-51, blaOXA-58, blaOXA-143, blaOXA-235.	Mediates carbapenem resistance by overexpression of OXA-23 and OXA-51. OXA-23, blaPER and blaTEM is responsible for cefiderocol resistance
Aminoglycoside-modifying enzymes	Genes conferring resistance are located on chromosomes, chromosomal genomic islands, plasmids, transposons or class I integrons	AME genes located in plasmids—aac3, aac6 family, aadA1, aadA2, aad A5, aadA13, aadA16, aph3,4 and 6 AME in Chromosome—aac (2′) Ib, aph (3″)Ib, Chromosomal genomic island -aph(6)Id	Aminoglycosides (Different enzymes have varying affinity to aminoglycosides)
Tetracycline-inactivating Monooxygenase	Plasmid	tet(X3), tet(X4), tet(X5)	Inactivates all tetracycline, including tigecycline, eravacycline and omadacycline
Macrolide 2-phosphotransferases	Plasmid	mph(A) and mph (E)	Erythromycin, azithromycin, clarithromycin

**Table 3:** Isolation of bacteriophages against *A. baumannii*.

S. No	Phage Name	Sample Source	Taxonomy of Phage	Bacteria	Reference
1	AB1I1L, AB1I1M, AB1I1P, AB1I1T, AB2I2, and AB2I3	Wastewater	Myoviridae	XDR *A. baumannii*	[110]
2	vB_AbaS_SA1	Hospital sewage	Sipho virus	MDR *A. baumannii*	[107]
3	Abgy202141	Underground sewage	Friunavirus	ESBL-producing *A. baumannii*	[111]
4	vB_AbaP_Indie	Wastewater treatment plants	Drulisvirus	MDR *A. baumannii*	[112]
5	T1245, T444, T515, T17, T92, P521, P1051, P1033, P245	Hospital sewage	Myoviridae: P105, P1033, P245; Podoviridae: T1245, T444, T515, T17, P521	MDR *A. baumannii*	[113]
6	vB-AbauM- Arak1	Urban wastewater	Myoviridae	XDR *A. baumannii*	[114]
7	vB_AbaP_ZC2 (ΦZC2) and vB_AbaM_ZC3 (ΦZC3)	Wastewater	Podovirus: ΦZC2 and Myovirus: ΦZC3	MDR *A. baumannii*	[115]
8	QAB 3.4	Sewage water samples	Not identified	XDR *A. baumannii*	[116]
9	vAbaIN10	Hospital wastewater	Friunavirus	CRAB	[117]
10	vB_MZM_2AB-P and vB_MZM_4AB-P,	Sewage samples	Caudoviricetes	MDR *A. baumannii*	[118]
11	vB_AbaP_W8, vB_AbaSi_W9, and vB_AbaSt_W16)	Sewage samples	Podovirus: vB_AbaP_W8; Myovirus: vB_AbaSi_W9, and vB_AbaSt_W16	CRAB	[119]
12	YZ2	Untreated wastewater	Friunavirus	CRAB	[120]
13	vB_AbaP_PhE54	Hospital sewage	Podoviridae	CRAB	[121]
14	vB_AbaM_ISTD, and vB_AbaM_NOVI	Belgrade wastewaters	Myoviridae	CRAB	[122]
15	vB_AbaS_AKO8a, vB_AbaS_PS118, vB_AbaS_B612, vB_AbaS_MCR, vB_AbaS_IDQ7, vB_AbaS_89P13, vB_AbaS_CRL20, and vB_AbaS_CIM23	Hospital sewage and wastewater	Kagunavirus	MDR *A. baumannii*	[123]
16	vB_AbaM_PhT2, vB_AbaM_PhT4, vB_AbaP_PhT29, vB_AbaP_PhT39, vB_AbaM_PhT44	Hospital wastewater	Myoviridae: vB_AbaM_PhT2, vB_AbaM_P hT4, vB_ AbaM_PhT44 Podoviridae: vB_AbaP_PhT29, vB_AbaP_PhT39.	*A. baumannii*	[124]
17	D2SVT	Water samples	Archaeoviruses	CRAB	[125]

NT: Not Tested; XDR: Extensive drug resistance; MDR: Multidrug resistance; ESBL: extended spectrum β-lactamase; CRAB: Carbapenem-resistant *Acinetobacter baumannii*.

## Data Availability

The original contributions presented in this study are included in the article. Further inquiries can be directed to the corresponding author(s).
